# A multidimensional approach to the resilience in older adults despite COVID-19

**DOI:** 10.1186/s12877-022-03472-y

**Published:** 2022-10-11

**Authors:** G. Perez-Rojo, J. López, C. Noriega, C. Velasco, I. Carretero, P. López-Frutos, L. Galarraga

**Affiliations:** 1 Department of Psychology and Pedagogy, School of Medicine, Universidad San Pablo-CEU, CEU Universities, 28925 Madrid, Spain; 2 Departamento de Psicología y Pedagogía, Facultad de Medicina, Universidad San Pablo-CEU, CEU Universities, Campus de Montepríncipe, 28925 Alcorcón, Madrid Spain; 3 Universidad Francisco de Vitoria, Universidad Francisco de Vitoria, 28223 Madrid, Spain

**Keywords:** Strengths, Protective factors, Crisis, Descriptive survey study

## Abstract

Researchers have mainly focused on aging risk factors and COVID-19 consequences. However, older adults have proved their ability to overcome adversities along their life. Resilience is a protective variable that dampens the impact of stress. Based on MacLeod’s et al. (2016) approach, we aimed to analyze the relationship between older adults’ resilience and COVID-19 related-stressors as well as their physical, mental, and social characteristics. Eight hundred eighty-nine people aged 60 and over participated in this study. Older participants, women, having better perceived health and not losing a loved one because of the virus were associated with more resilience. Moreover, higher levels of gratitude, personal growth, life purpose and lower levels of depression were associated with greater scores in resilience. This study offers a change of perspective in which aging is perceived from a positive viewpoint by focusing on easily accessible resources that may help older adults to cope with adverse situations.

The COVID-19 pandemic is a highly uncontrollable stressful situation [1] with potential long-term consequences on physical and psychological well-being [2]. Although there are some studies that conclude younger people were more emotionally affected by COVID 19 pandemic than older people [3–6]. On the contrary, other studies show that older people suffered a bigger threat [7–10] and the case-fatality from COVID-19 was higher in population aged 65 and above [11]. And some authors found a relationship between emotional well-being and resilience and social support in family caregivers exposed to social isolation [12]. For this reason and the fact that traditionally aging has been linked to negative characteristics, most COVID-19 research has focused on older adults’ negative consequences [13]. However, not the whole group of older adults has been affected negatively. Several biopsychosocial variables could play an important role as protective factors [14]. One of the most remarkable aging characteristics is its heterogeneity [15], so age is not enough criterion for predicting the direct impact of the virus. What is more, difficult situations older adults have faced along their lives may have strengthened them because of developing protective psychological resources, like resilience [16].

According to the American Psychological Association, resilience is the process of adapting well when facing adversity, trauma, tragedy, threats, or significant sources of stress [17]. This consideration of resilience as a dynamic process has been generally accepted. Despite the increasing recognition of resilience like a key construct across the adult life span, the study of this phenomenon in older adults has flourished rather recently [18]. Research results suggest that older adults have the capacity for high resilience despite age-related changes (role changes, illnesses, loneliness, and grief) [19]. And, on the one hand, other author found that older people with higher resilience and greater capacity to handle unforeseen circumstances might make their well-being better despite adversities related with COVID-19 [20]. On the other hand, other researches found results in the same line and described how resilience helped them to cope and adapt to this crisis [21]. Finally, others also pointed out the importance of older adults’ resilience during the pandemic from a socioecological approach and considered important resources to help their communities [22]. Resilience has been considered as a multidimensional construct compounded by mental, social and physical factors and it is positively linked with successful aging and longevity [19]. According to a meta-analysis carried out, successful aging is a multidimensional construct, compounded by four dimensions: “avoiding disease and disability, having high cognitive, mental and physical function, being actively engage in life and being psychologically well adapted in later life” [23] (p. 664). Regarding physical characteristics, several studies have found higher resilience in women stressing the idea that women have to face with more difficult situations along their lives which may strengthen and empower them [24] or because the female concept of resilience is related to the character of the circumstances that are causing the adversity and the history of stress [25], despite they are more prone to be more frail and have poorer health [26]. Moreover, a study mentioned before, showed that resilience was adequate in times of pandemic in female family caregivers [12]. In contrast, very recent studies found worse resilience in women facing COVID-19 [27, 28]. And even, other study did not find a relationship between sex and resilience [29]. Other studies have suggested the association among resilience and better health, lower rate mortality and higher longevity even in the oldest old [30, 31].

Within mental characteristics, it was found a significant positive relationship among resilience and optimal outcomes, such as psychological well-being, gratitude or acceptance. When facing life challenges, many people can find a sense of purpose and meaning and even to develop one’s potential [16]. Furthermore, some research found a relationship between suffering and positive meaning and gratitude [32]. Gratitude is one of the character strengths related to the virtue of transcendence which determines a vital element of human existence [33, 34]. It is firmly associated with well-being, including personal growth, life purpose and self-acceptance [35]. What is more, other studies found that gratitude was the most effective emotion that increases after important adversities [36, 37]. And there is also a tendency that shows how people who feel and express gratitude manifest better physical and psychological health [38].

Adaptative coping strategies, like acceptance, can also help older adults to adapt gradually to daily challenges and are critical to recovering from stressful events. On the contrary, these variables show a negative relationship with emotional distress (depression and anxiety) [39]. Finally, contextual variables like warmth, support and family cohesion contribute to resilience [19]. However, there are some groups of people that are more vulnerable, and it is more difficult to them to cope with the trials of life, for example, people with socio-economic difficulties [40]. According to one study, the promotion of resilience is one of the possible practices of decreasing social and economic inequality [41]. And other found that even though some participants presented socio-economic inequality, their resilience was nevertheless reasonably elevated [40].

This study aimed to assess physical, psychological, and social factors related to resilience from a multidimensional approach. To our knowledge, although there are several studies about aging and COVID-19 considering a strengths-based approach, there is still a higher percentage of studies focused on older adults’ negative consequences and risk factors..

## Method

### Participants

We used snowball sampling to recruit 889 people community-dwelling aged 60 and over from Spain 3 weeks following the lockdown restrictions (from April to June 2020). The mean age was 68.45 years old (SD = 6.06). 4.6% of participants aged 80 years and older. We received responses from 937 respondents but 889 finally participated (94.87% response rate). Forty-eight participants were excluded because they were under 60 years old. Most participants were women (62%), were living in their own home (92%) with their spouse or partner (62.5%) and reported a good (43.5%) or fair (34.1%) perceived health. 76 (8.5%) participants had COVID-19 symptomatology; 9 (1.01%) had been hospitalized, 170 (19.12%) had a close family member or friend who has been hospitalized and 93 (10.46%) reported the loss of a loved one by the virus.

### Measures

We prepared an assessment protocol that included questions about sociodemographic characteristics, self-perceived health, and characteristics of the COVID-19 lockdown situation. Moreover, some public domain standardized questionnaires were used:

- Brief Resilient Coping Scale (BRCS) [42]. The objective of this scale is to assess “the ability to bounce back or recover from stress” [43]. This scale comprises 4 items to measure the preference to handle with adversities in a strongly adaptive management, ranging from 1 = strongly disagree to 5 = strongly agree. And item sample is “I look for creative ways to alter difficult situations “. In our sample it had an adequate internal consistency (Cronbach’s α = .791).
- The Family APGAR [44]. We used this scale in order to assess family functioning (adaptability, partnership, growth, affection and resolve). We used the total score of this scale. It is a 5-item Likert scale with 3 response options (almost always, some of the time and hardly ever). An item example is “I am satisfied that I can turn to my family for help when something is troubling me”. The higher the score, the greater the indication of a functional family system. From 7 to 10 points implies a functional family (FF), from 4 to 6 points mild family dysfunction (MFD), from 0 to 3 points severe family dysfunction (SFD). It was found and adequate internal consistency in our study (Cronbach’s α = .780).
- Gratitude subscale of the Values in Action Inventory of Strengths-Short Form [45]. It comprises 5 items and each of them is rated on a 5-point Likert scale and it was used to evaluate gratitude, described as being conscious and grateful for the good things that take place. An item example is “I am an extremely grateful person”. In our research it showed good reliability (Cronbach’s α = .841).
- The Acceptance and Action Questionnaire – II (AAQ-II) [46]. We used this reliable and valid instrument composed by 7 items with 7 response options from 1 (very inadequate to describe myself) to 7 (very adequate to describe myself) to measure experiential avoidance. An item example is “My painful experiences and memories make it difficult for me to live a life that I would value”. In our sample it was found good internal consistency (Cronbach’s α = .890). We used the opposite pole of acceptance because both terms are used frequently in the literature and refer to the same underlying construct. Indeed, the lower score the greater acceptance.
- Ryff’s Psychological Well-Being Scales. We used two scales, personal growth (the extent to which they were making use of their personal talents and potential) and purpose in life (the extent to which respondents felt their lives had meaning, purpose and direction) assessed by 7 and 6 items respectively, to measure psychological well-being because Ryff’s considers them the core elements to psychological wellbeing. An item example of personal growth is “For me, life has been a continuous process of learning, changing, and growth” and for purpose in life is “I enjoy making plans for the future and working to make them a reality”. Both scales were scored in a 7-point Likert [47]. It was found an adequate internal consistency for personal growth (Cronbach’s α = .625) and purpose in life (Cronbach’s α = .808).

### Procedure

A cross-sectional web-based survey was carried out through social media (Twitter, WhatsApp, LinkedIn). The validity and reliability of Internet surveys for analyzing subjective well-being are comparable to paper-based versions [48]. Participation was voluntary and no reward was given. Informed consent was obtained from all respondents, and confidentiality was explicitly guaranteed. The Ethics Committee of Universidad San Pablo-CEU approval the study (code approval: 436/20/26). We used the STROBE checklist recommendations to strengthen the reporting of observational studies in epidemiology.

## Results

First,, we calculated the level of resilience of this sample using the cut-off purposed by one study: scores from 4 to 13 low resilience, scores from 14 to 16 medium resilience and scores from 17 to 20 high resilience [42]. We found that 43.4% presented high resilience 35.5% medium resilience and 21% low resilience.

Based on a resilience approach [19], the independent variables included in a linear regression model were the following: physical, COVID-19 related-stressors, mental and social characteristics. The variables related with sociodemographic and physical characteristics (age, sex and perceived health) were entered as a block in a first step; in a second step, controlling the effect of the first block variables, we introduced the COVID-19-related-stressors (fear to the virus, presence of symptomatology, being hospitalized, having a close family member or friend who has been hospitalized and losing a loved one by the virus); in a third step, controlling the effect of the previous ones, the variables associated with mental characteristics were introduced as a block (experiential avoidance, gratitude, personal growth, life purpose, anxiety and depression); and finally, in the fourth step, we included the variables related with social characteristics (family functioning).

In Table 1 are the descriptive statistics for the assessed variables.

**Table 1:** Descriptive statistics

Variable	Mean ± S.D.	Range
Age	68.45 ± 6.06	60–95
Resilience	15.78 ± 3.30	4–20
Personal growth	28.25 ± 4.38	13–41
Life purpose	27.89 ± 4,77	8–36
Avoidance experience	19.65 ± 6.77	7–49
Gratitude	22.21 ± 2.94	9–25
Family functioning	8.75 ± 1.85	0–10
Anxiety	5.13 ± 3,24	0–18
Depression	3.64 ± 2.82	0–17

The explained variance of resilience significantly increased in every step (${R}_{Step\ 1}^2$ =.066; ${R}_{Step\ 2}^2$ =.086; ${R}_{Step\ 3}^2$ =.108; ${R}_{Step\ 4}^2$ =.196), but the main differences in the variance explained by the predictors can be found the fourth step (∆R^2^ = .196). The results of the final linear regression model can be found in Table 2. This linear equation model explained 30.4% of the total resilience variance. All physical variables significantly contributed to explaining resilience. Participants who were older, female and those who indicated better perceived health also showed more resilience. Among the COVID-19-related-stressors variables, only losing a loved one was related to less resilience. Regarding mental characteristics, higher levels of gratitude, personal growth, life purpose and lower levels of depression were associated with greater scores in resilience.

**Table 2:** Results of the final linear regression model to predict resilience scores

Variable	β	SE	_s_β	t	p
Constant	3.83	1.87	–	2.05	.04
Age	.05	.02	.08	2.77	<.01
Sex (Men = 2)	−.58	.20	−.09	−2.95	<.01
Perceived health	.31	.13	.08	4.44	<.05
Fear to virus	−.18	.12	−.05	−1.45	.15
Presence of symptomatology	.01	.36	.00	.02	.98
Being hospitalized	−1.74	1	−.05	−1.74	.08
Close family member or friend hospitalized	.41	.27	.05	1.55	.12
Losing a loved one	−.71	.34	−.07	−2.06	<.05
Family functioning	−.01	.06	−.00	−.09	.93
Experiential avoidance	−.01	.02	−.02	−.42	.68
Gratitude	.12	.04	.11	3.44	<.01
Personal growth	.08	.02	.10	3.01	<.01
Life purpose	.18	.03	.26	7.36	<.01
Anxiety	−.07	.04	−.06	−1.53	.13
Depression	−.18	.05	−.15	−3.71	<.01

All variance inflation factor (VIF) values for the independent variables were below 10 (range of 1.003–2.092) and all tolerance statistic values are over 0.2 (range of 0.464–0.988).

## Discussion

Most information regarding older adults’ psychological impact during the COVID-19 pandemic is based on risk factors. This study stresses the role played by protective factors considering the resilience multidimensional approach proposed by some authors that entails physical, COVID-19 related-stressors, mental and social factors 3 weeks following the lockdown restrictions [19].

This work points out that each group of variables obtain a differentiate explicative power, explaining a total of 30.4% of the resilience variance. The variables which more contributed to predicting it included: age, female and those who indicated better perceived health (physical characteristics), losing a loved one (COVID-19-related-stressors variables) and higher levels of gratitude, personal growth and life purpose and lower levels of depression (psychological characteristics). The results of this study that perceived health and resilience have the same relationship before and after pandemic situation [49]. These results may be also related to the conceptual overlap among these variables. However, while resilience may be considered the result, in the ability to overcome adversities, the other variables could play the role of increasing the resilience levels. These results support the hypothesis that resilience results from a combination of multiple characteristics [16, 24, 30, 32, 39]. However, it would be interesting to control the role of sex, like a relevant influencing variable, in these results.

Our study also supports a positive perspective of ageing by showing high levels of resilience in a high percentage of older adults. This, in line with recent literature [12, 20–22] recognizes the importance of older adults‘strengths. We observed that psychological resources like resilience, gratitude, personal growth, and life purpose may have empowered older adults to handle very severe adversities along their lives, included the current pandemic situation, confronting the idea that “older adults are more vulnerable” [50]. As a result, these variables could be considered in future intervention programs with older people that aim to increase their well-being levels. All of these variables could be trained from the positive psychology approach to build a stronger mental health that buffers against adversities and promotes well-being according to different studies with healthy older people [51–53] and with older people with long-term conditions [54].

Although some studies found more depression, for example in younger people [3–6], others found that older people are more affected by the pandemic [7–11]. But these results cannot support the idea of age as a weakness by itself. However, it is true that the older the more probability to have negative consequences like financial insecurity or depressive symptomatology. The stressors across the life course that older adults must face might train them and provide an opportunity to build resilience and age successfully. This could help to eliminate aging negative stereotypes and attitudes. In this line, resilience could be a variable that may buffer the impact of future adverse events [55].

Although this research has generated interesting findings, some limitations need to be addressed. First, this is a cross-sectional study that does not allow the possibility to set temporal precedence and establish causal relationships. Longitudinal studies are required to ensure the stability of these results. Furthermore, it is based in a convenience and nonprobability sample which might not be representative of the whole Spanish population of 60 years and over. In addition, none of participants lived in nursing homes and the assessment of resilience in this group is relevant because the personal and environmental circumstances are different to community dwelling older people. Although women life expectancy is higher than men, women are overrepresented in this study and a wider range of men sample is needed. In addition, the are some variables not explored in this study, like religion [56], chronic medical illness [57] and economic security [58]. Moreover, we did not include established QoL indicators, and the internal consistency of personal growth is weak and for this reason the results could be not generalized. Perhaps for this reason, although the explained variance increased after each step, the overall explained variance is not that high. This could indicate that more influencing variables remain to be identified. Indeed, results of this study are not generalizable to other countries with a different well-being system, for example developing countries. Another drawback to consider is that we do not use ANOVAS in terms of age cohorts or sex for example. Finally, the sample consisted of non-institutionalized people. These results could be not representative of the older adults living in long-term care facilities. Nevertheless, older Spanish adults’ dwells in the community.

To conclude, this study provides a shift in the aging image, focusing on the importance of resilience as a dynamic process that helps to promote the process of successful aging and longevity [59, 60]. It also emphasizes the need for developing policies and preventive and intervention programs that promote older adults’ resilience and the associated variables that, in turn, may buffer the impact of adversities on their physical and psychological health. This pandemic situation has demonstrated how technology could help in different settings included the care of older people. There are some online psychological interventions that have shown their efficacy on older people before and during the pandemic situation. For example, internet cognitive behavior therapy was tested, and it was useful and cost-effective [61], and also helped to treat insomnia [62] and even avoid the spread of the infection during the pandemic situation [63]. Moreover, the use of life-story review can reduce depression in older adults and can help them to review their life, providing an emotional catharsis and strengthen resilience [58]. However, the digital divide in older people should be solved to assure the accessibility to online interventions [20]. Moreover, the use of life-story review can reduce depression in older adults and can help them to review their life, providing an emotional catharsis and strengthen resilience [58]. Finally, it would be very useful if resilience could be assessed along their lives and these programs were designed in collaboration with the older adult [64].

## Data Availability

The datasets generated and/or analysed during the current study are available from the corresponding author on reasonable request.
